# Theory, simulation and experimental results of the acoustic detection of magnetization changes in superparamagnetic iron oxide

**DOI:** 10.1186/1471-2342-11-16

**Published:** 2011-06-29

**Authors:** Bernhard Gleich, Jürgen Weizenecker, Jörn Borgert

**Affiliations:** 1 Tomographic Imaging Group, Philips Technologie GmbH Innovative Technologies, Research Laboratories, Röntgenstraße 24-26, 22335 Hamburg, Germany; 2 Department of Electrical Engineering, University for Applied Sciences, Moltkestraße 30, 76133 Karlsruhe, Germany

## Abstract

**Background:**

Magnetic Particle Imaging is a novel method for medical imaging. It can be used to measure the local concentration of a tracer material based on iron oxide nanoparticles. While the resulting images show the distribution of the tracer material in phantoms or anatomic structures of subjects under examination, no information about the tissue is being acquired. To expand Magnetic Particle Imaging into the detection of soft tissue properties, a new method is proposed, which detects acoustic emissions caused by magnetization changes in superparamagnetic iron oxide.

**Methods:**

Starting from an introduction to the theory of acoustically detected Magnetic Particle Imaging, a comparison to magnetically detected Magnetic Particle Imaging is presented. Furthermore, an experimental setup for the detection of acoustic emissions is described, which consists of the necessary field generating components, i.e. coils and permanent magnets, as well as a calibrated microphone to perform the detection.

**Results:**

The estimated detection limit of acoustic Magnetic Particle Imaging is comparable to the detection limit of magnetic resonance imaging for iron oxide nanoparticles, whereas both are inferior to the theoretical detection limit for magnetically detected Magnetic Particle Imaging. Sufficient data was acquired to perform a comparison to the simulated data. The experimental results are in agreement with the simulations. The remaining differences can be well explained.

**Conclusions:**

It was possible to demonstrate the detection of acoustic emissions of magnetic tracer materials in Magnetic Particle Imaging. The processing of acoustic emission in addition to the tracer distribution acquired by magnetic detection might allow for the extraction of mechanical tissue parameters. Such parameters, like for example the velocity of sound and the attenuation caused by the tissue, might also be used to support and improve ultrasound imaging. However, the method can also be used to perform imaging on its own.

## Background

Magnetic Particle imaging (MPI) is a novel method for medical imaging. It can be used to measure the local concentration of a tracer material based on iron oxide nanoparticles. While the resulting images show the distribution of the tracer material in phantoms or anatomic structures of subjects under examination, no information about the circumjacent tissue is being acquired. To expand MPI into the detection of soft tissue properties, a new method is proposed, which detects acoustic emissions caused by magnetization changes in superparamagnetic iron oxide. These signals may allow for the in vivo determination of soft tissue parameters, such as attenuation and velocity of sound. This article presents an introduction to acoustically detected MPI, a comparison to magnetically detected MPI, as well as first simulations and experimental results.

Magnetic Particle Imaging (MPI) determines the presence of iron oxide nanoparticles from their nonlinear magnetization behavior [1]. Such nanoparticles are well known as superparamagnetic iron oxide (SPIO). They are available as a clinically approved contrast agent for liver examinations in magnetic resonance imaging (MRI) [2] and are usually administered into the bloodstream via intravenous injection. In the context of MPI, such nanoparticles are used as a tracer material. To examine the magnetization properties of the tracer, MPI deploys a field free point (FFP) realized in a strong gradient field, called the selection field. This field free point is moved across the sample or phantom to gather information about the distribution and concentration of the tracer material. Using additional homogeneous magnetic fields, called drive fields, this movement can be realized in a very fast manner, leading to dynamic 2D [3] or 3D [4] imaging. The very signal generation takes place when the FFP passes a point in space containing tracer material and the magnetization of the nanoparticles is changed. This change in magnetization results in an induced signal that can be picked up in a recording coil. Depending on the steepness of the magnetization curve and the strength of the selection field, information about the distribution and concentration of the tracer material can be obtained with high spatial resolution. To reconstruct an image from this information, the properties of the instrumentation, e.g. coil sensitivities, and the characteristics of the tracer have to be determined. This information is usually encapsulated into the so-called system function. Practically, the system function can be determined by measuring the MPI signal generated by a small sample at a sufficient number of points within the field of view. In a recent paper [5] it was shown, that for the one-dimensional case the system function can be composed of Chebyshev polynomials of the second kind, assuming idealized particles.

**Figure 1:**
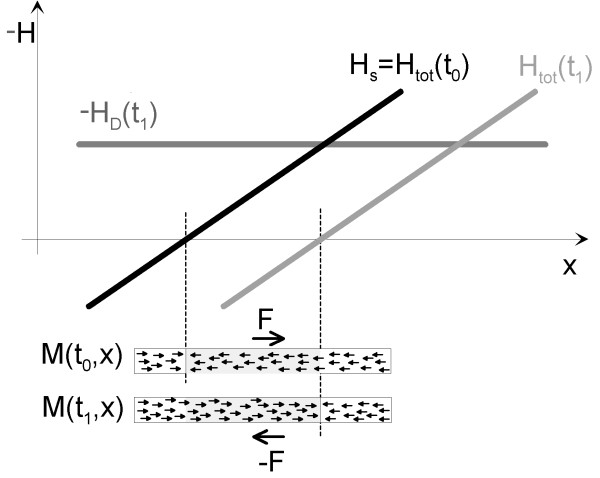
**One-dimensional signal generation in MPI**. The signal formation in acoustically detected magnetic particle imaging bears a lot of similarity to the magnetic case. Assuming that the drive field is zero at a time t0, the total field Htot is given solely by the selection field Hs. As the magnetization M of the particles always points in the same direction as the magnetic field, the magnetization of the particles being left or right of the FFP points in opposite directions. The force always points away from field free point (FFP). Consequently,. when the FFP is moved along the sample by the drive field HD(t1), the magnetization for some of the particles changes from pointing left to pointing right. As the selection field exerts a force on the magnetization, the change in magnetization is accompanied by a change in force. A change in force in a material generates a sound wave, which can be detected with appropriate microphones. Figure 2 - Another sample figure title

Acoustically detected MPI involves the same basic components as in the magnetic case, i.e. a selection field to form a strong gradient including the field free point and a drive field to move the field free point across the volume of interest. The only additional component is a microphone, which is used to detect the sound emission, and which has to becoupled to the examined object, e.g. a suitable phantom. To illustrate the signal generation in acoustically detected MPI, the examination of the one-dimensional case is sufficient, as shown in figure 1. When a magnetic particle is placed in the selection field, a force is acting on it. As the magnetization of the particles points in field direction, the force will be directed away from the FFP. If the FFP passes the particle, which is caused by the drive field, the direction of the force changes. This change in force can be measured as a sound wave.

In the following, the achievable spatial resolution is estimated in comparison to magnetically detected MPI. Assuming that the magnetic particles behave in accordance to Langevin theory as described in [6,7], the force on the magnetic material is given by(1)

Here, M0 is the saturation magnetization of the material, L the Langevin function, μ is the magnetic moment of the particles, and kBT the thermal energy. If the particles are in saturation, which is the case if the FFP is sufficiently far away, the above formula can be simplified to(2)

This means that the force points in the direction of the strongest change of magnitude. For the simplified case of one-dimensional signal generation, equation 1 simplifies to:(3)

with α being an abbreviation for μ/kBT and B being the magnitude of the field in x-direction, i.e. B = |Bx|, The value of μ depends on the particle volume and thus on the particle diameter. Gx is dBx/dx, the gradient of the Bx component, which can assumed to be constant for the region of interest.

**Figure 2:**
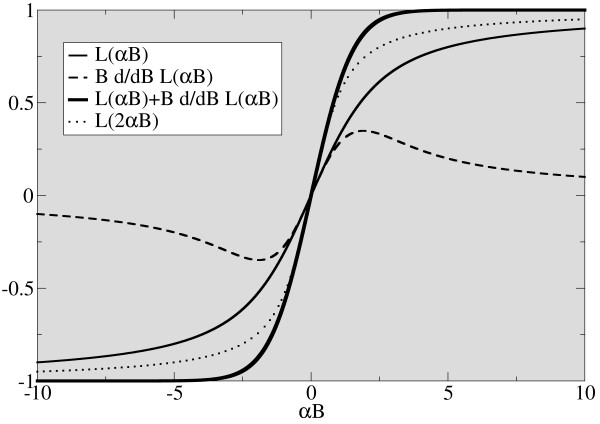
**Resolution-determining terms in magnetic and acoustic MPI**. The individual components of equation 3 are displayed as function of the product of particle parameters and magnetic field Saturation is reached for much lower fields in the acoustic case (bold curve) as in the magnetic L(αB) case. A comparison of the acoustic with a magnetic curve assuming doubled α (dotted line) indicates more than twice the resolution.

In magnetically detected MPI, the flux through the receiving coil is proportional to the magnetization, i.e. L(αB). As is apparent from equation 3, the corresponding term for the detection of a change in force in acoustically detected MPI is  In comparison to L(αB), this term has twice the steepness for small values of B, c.f. figure 2. For large B, the limit of both terms is given by L(αB), i.e. 1. Assuming the same signal-to-noise ratio for both methods, the theoretical resolution in acoustically detected MPI is twice the resolution achievable with magnetically detected MPI for given particle properties α.

**Figure 3:**
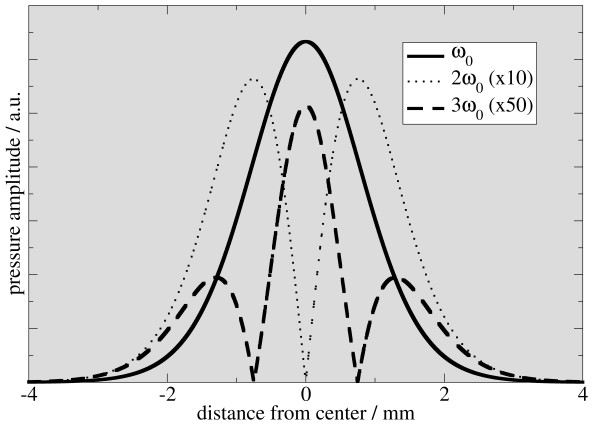
**Simulated acoustic MPI system function in frequency space**. At each position × of the infinitely small sample, the system response in the time domain is calculated and Fourier-transformed. Fourier-amplitudes are plotted for the first 3 harmonics. A value of 5 T μ0-1/m was assumed for the gradient strength G×, the drive field amplitude was set to 2.5 mT μ0-1, and a particle diameter of 17 nm was assumed. Figure 1 - One-dimensional signal generation in MPI.

As a next step, the impact of the system function will be analyzed. The definition of the system function in the acoustic case is in complete analogy to the magnetic case. The system function encapsulates information about the particle dynamics, the coil sensitivities and the movement of the FFP caused by the drive field. It is attained by measuring the MPI signal of a small sample for a set of positions covering the region of interest or field of view. A later measurement of an unknown tracer distribution can be decomposed into a superposition of the individual signals measured during system function acquisition. The weighting coefficients for the individual signals represent the concentration of the tracer at the very position at which the individual signals have been recorded [1]. Mathematically, the system function can be obtained by replacing Bx by Gxx+Asin(ω0t) in formula 3. The amplitude A and the frequency ω0 are the parameters describing the drive field. In figure 3, the magnitudes of the first three harmonics of the Fourier-transformed system functions are plotted. It is obvious that the components of the system function are similar to the case of magnetic detection, albeit they do not resemble Chebyshev-polynomials, as described in [6].

Finally, the sensitivity for acoustically detected MPI is analyzed. For that purpose, the generated pressure has to be compared to the noise pressure. The lowest possible noise pressure is generated by thermal fluctuations. According to [8], it can be expressed as:(4)

with A being the detector area and Δ*f *being the bandwidth.  is the real part of the sound impedance, which is given by the product of density and speed of sound, as long as the damping is negligible. Neglecting the damping is justified for all tissues that allow a penetration of the sound wave of more than one wavelength. For a square millimeter sized detector and material properties of water at 310 K, the noise pressure is . The signal generation by magnetic particles can be estimated by assuming a cube of 1 mm^3^ filled with 0.5 mol(Fe)/l present as magnetite, leading to a saturation magnetization of approximately 4.4 mT μ0-1. In a gradient field of 5 T μ0-1/m this translates into a force of 18 μN or a pressure of 18 Pa and results in a signal-to-noise ratio of 1.1 × 105 for a measuring time of one second. A reasonable detection limit would be 22 μmol(Fe)/l, which corresponds to five standard deviations and should be detectable within one second. For comparison, a typical dosage of the applied material in MRI is 8 to 40 μmol(Fe)/l [2] with a detectable local concentration of about 50 μmol(Fe)/l [9]. In MPI, an ultimate detection limit of about 20 nmol(Fe)/l has been estimated [1].

## Methods

**Figure 4:**
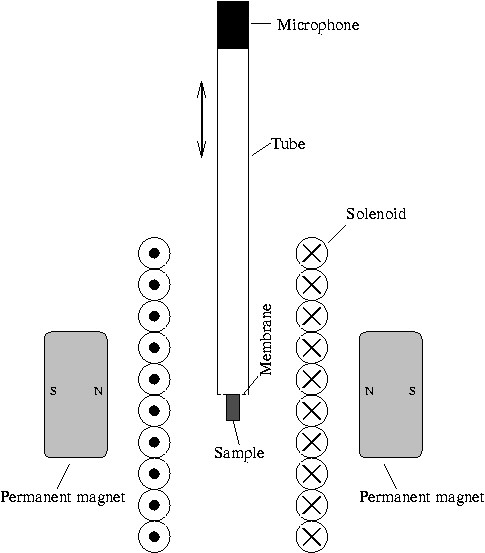
**Experimental set-up for acoustically detected MPI**. Permanent magnets made from FeNdB produce a selection field of 5 T μ0-1/m. A solenoid produces a drive field of 2.5 mT μ0-1. The sample of magnetic material is glued to a thin and flexible polymer membrane and attached to a tube. A microphone is mounted to the other end of the tube. The tube with sample and microphone is mounted to a robot to move the sample to defined positions.

A schematic of the experimental acoustic MPI device is sketched in figure 4. Permanent magnets made from FeNdB with a diameter of 20 mm and a height of 10 mm produce a gradient of 5 T μ0-1/m in vertical direction. The drive field coil (length 35 mm, diameter 22 mm) is a single layer solenoid connected to an audio amplifier and resonantly matched. The drive field amplitude is 2.5 mT μ0-1 at a frequency of 700 Hz. Using a frequency of 700 Hz ensures that the microphone used operates within its optimal sensitivity range and moreover, that the resulting acoustic emissions is audible. The sample of magnetic material, undiluted Resovist (0.5 mol(Fe)/l; Bayer Schering Pharma, Leverkusen), is filled into a bore with a diameter of 1.5 mm and a length of 2 mm. The particle size distribution shows a maximum at small particles, yet, large particles are also present [1]. The sample is glued to a flexible cling film membrane, which is attached to a plastic tube with an inner diameter of 6.3 mm and a length of 390 mm. At the end of the tube a calibrated microphone (Roga instruments, type RG-50, frequency response 0.5 dB at the used frequencies) is attached using a rubber tube as an adaptor. The electrical output of the microphone is connected to a spectrum analyzer (Rhode & Schwarz, FSEA). The plastic tube with sample and microphone was attached to a robot (Isel, Flachbettanlage 1) to move the sample in vertical direction. To optimize the measurement, the sample was positioned such that the recorded signal in the fundamental frequency was maximized. Initially, it was displaced 3.25 mm upwards and subsequently moved downwards with an 0.125 mm stepping to cover a whole 6.5 mm. For each step, the amplitudes of the first three harmonics were recorded. Therefore, the bandwidth of the spectrum analyzer was set to 2 Hz and the measurement was averaged over ten individual recordings.

## Results

**Figure 5:**
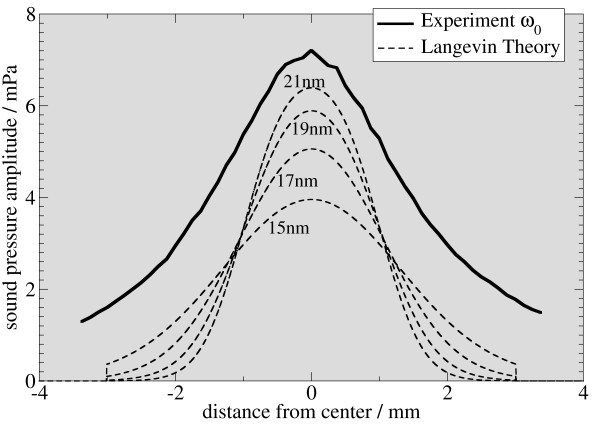
**Comparison of experimental and simulated data**. Using a simulation scheme as in figure 3, a superposition of the response calculated from simulated system functions and the experimental data is plotted for the first three harmonics (this figure: 1st harmonic) for nano-particles of different sizes. The best fit is observed for particles between 15 and 17 nm. For the fundamental frequency, the 15 nm particles yield optimal signal match, whereas for the third harmonic, the 17 nm particle data is in good agreement with the simulation.

**Figure 6:**
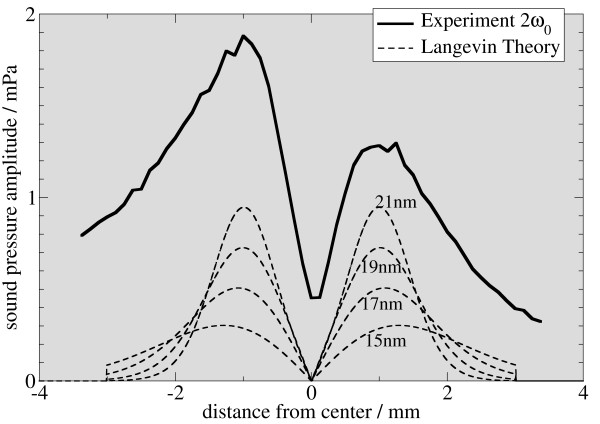
**Comparison of experimental and simulated data**. Using a simulation scheme as in figure 3, a superposition of the response calculated from simulated system functions and the experimental data is plotted for the first three harmonics (this figure: 2nd harmonic) for nano-particles of different sizes. The best fit is observed for particles between 15 and 17 nm. For the fundamental frequency, the 15 nm particles yield optimal signal match, whereas for the third harmonic, the 17 nm particle data is in good agreement with the simulation.

**Figure 7:**
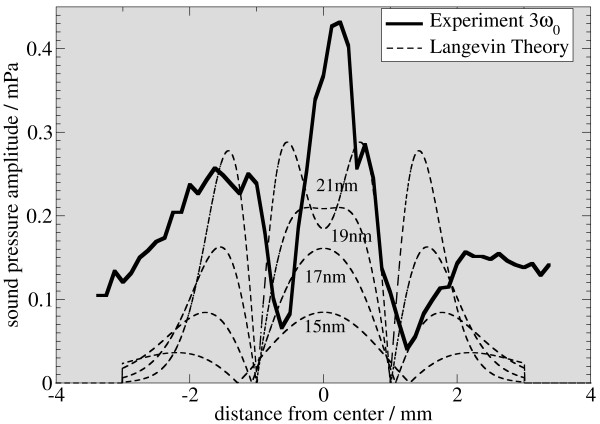
**Comparison of experimental and simulated data**. Using a simulation scheme as in figure 3, a superposition of the response calculated from simulated system functions and the experimental data is plotted for the first three harmonics (this figure: 3rd harmonic) for nano-particles of different sizes. The best fit is observed for particles between 15 and 17 nm. For the fundamental frequency, the 15 nm particles yield optimal signal match, whereas for the third harmonic, the 17 nm particle data is in good agreement with the simulation.

In figure 5, 6, and 7, the signal intensity of the first three harmonics is plotted as a function of the vertical displacement. For comparison, simulated data for different particle sizes is added to the graphs. The simulated data is derived from the system function in figure 3. Therefore, an average is calculated from a sliding window, whose length is the same as the sample length of 2 mm. Experimental and simulated data are in reasonable agreement with respect to the shape of the graph and the location of the minima and maxima, the graphs showing the same features for particle sizes from 15 to 17 nm.

## Discussion

There are some remarkable differences between simulation and experiment. Most notably, the symmetry is broken in the experimental data. This may be attributed to a small tilt of the sample with respect to the vertical direction. Additionally, the membrane can exhibit an asymmetric stiffness, caused by the gluing process. Together with off-axis components of the force acting on the membrane, this can break the symmetry. A background signal can be the cause of symmetry breaking for the second harmonic. This background signal can change the relative height of the peaks in figure 5, 6, and 7, depending on its relative phase. The presence of such a background in the experiment is evident, as the measured amplitude never drops to zero.

Since the microphone used for the measurements is calibrated, the recorded signal can be transformed to sound pressure values. The fundamental frequency shows a peak amplitude of approximately 7 mPa. From the amount of magnetic material and the diameter of the tube, a pressure of 2 Pa is expected, i.e. a factor 300 higher. This discrepancy might be attributed to the fact that the particles in Resovist do not reach saturation at 2.5 mT μ0-1 [1], as most of the particles seem to be very small, i.e. below ten nm [10]. As a consequence, only a small fraction of the particles contribute to signal generation. Additionally, the membrane itself might be a cause for the discrepancy. During a change in pressure, the tension of the membrane counteracts the force on the membrane.

## Conclusions

Using an experimental magnetic particle imaging setup, it was possible to detect acoustic emissions of a magnetic tracer material. A theoretical model was applied to simulate and predict the spatial distribution of the signal. The experimentally detected sound amplitudes were in good agreement with the simulations.

For medical applications, the information gained from acoustic detection might be used to generate an additional contrast to magnetically detected MPI images, which only show the distribution and concentration of the tracer. A well-defined sound wave is emitted from the position of the field free point (FFP) and travels through the tissue to the detector. During this propagation, the wave is attenuated, scattered, and delayed. As a consequence, it should be possible to image mechanical tissue properties, such as sound velocity and attenuation, which are not directly accessible to ultrasound imaging.

As an additional benefit, these tissue properties would be useful to improve image quality in ultrasound imaging by eliminating multi-scattering artefacts. For these types of applications to be feasible, it would be necessary to realize an efficient mechanism of sound generation from the FFP. If the experiment described herein would be performed in tissue, the signal would probably be very low, as the pressure rapidly decreases with distance from the FFP. The main reason for this is destructive interference between positive and negative pressure waves, as observed in box-less loudspeaker designs. This effect may be circumvented by moving the FFP faster than the local sound velocity, analogous to the Cerenkov Effect [11] for charged particles which are moving faster than light in the given medium.

## Competing interests

BG and JB are employees of the Philips Technologie GmbH Forschungslaboratorien, Hamburg, Germany. JW has been an employee of the Philips Technologie GmbH Forschungslaboratorien, Hamburg, Germany at the time the research had been conducted. He is now full time professor at the Department of Electrical Engineering, University of Applied Science, Karlsruhe, Germany and a part time consultant to his former employer. The authors hold several patents on Magnetic Particle Imaging and related technologies.

## Authors' contributions

BG and JW derived the basic concept of acoustically detected MPI and the mathematical treatment, as well as conducted the experiments. JB coordinated the research and supported the work in helpful discussions. The manuscript was written jointly and approved by all three authors.

## Pre-publication history

The pre-publication history for this paper can be accessed here:

http://www.biomedcentral.com/1471-2342/11/16/prepub
